# Infant neural sensitivity to eye gaze depends on early experience of gaze communication

**DOI:** 10.1016/j.dcn.2018.05.007

**Published:** 2018-05-26

**Authors:** Angélina Vernetti, Nataşa Ganea, Leslie Tucker, Tony Charman, Mark H. Johnson, Atsushi Senju

**Affiliations:** a Centre for Brain and Cognitive Development, Birkbeck, University of London, Malet Street, London WC1E 7HX, UK; b Department of Psychology, Institute of Psychiatry, Psychology & Neuroscience, King’s College London, UK; c Department of Psychology, University of Cambridge, UK

**Keywords:** Eye gaze, Event-related potential, Infant study, Social experience, Non-verbal communication

## Abstract

A fundamental question in functional brain development is how the brain acquires specialised processing optimised for its individual environment. The current study is the first to demonstrate that distinct experience of eye gaze communication, due to the visual impairment of a parent, affects the specificity of brain responses to dynamic gaze shifts in infants. Event-related potentials (ERPs) from 6 to 10 months old sighted infants with blind parents (SIBP group) and control infants with sighted parents (CTRL group) were recorded while they observed a face with gaze shifting *Toward* or *Away* from them. Unlike the CTRL group, ERPs of the SIBP group did not differentiate between the two directions of gaze shift. Thus, selective brain responses to perceived gaze shifts in infants may depend on their eye gaze communication experience with the primary caregiver. This finding highlights the critical role of early communicative experience in the emerging functional specialisation of the human brain.

## 1 Introduction

From birth, infants show a remarkable capacity to detect and process the eye gaze of others. Newborns preferentially orient to faces making eye contact (Batki et al., 2000; Farroni et al., 2002), and shift their attention to the direction of perceived gaze shift (Farroni et al., 2002). Newborns preference for face-like pattern also involves detecting darker elements against lighter background (Farroni et al., 2005), which could be optimised to detect human eyes, characterised by a darker iris against white sclera (Gliga and Csibra, 2007). As eye gaze is a key channel of non-verbal communication in humans (Kleinke, 1986), such an early-emerging predisposition to process eye gaze is adaptive, preparing infants for social and communicative learning from parents and other adults (Csibra and Gergely, 2009).

Recent evidence suggests that this newborns’ predisposition is followed by brain adaptation to the individual’s specific sociocultural environment, which may vary in degree of exposure to communicative eye gaze. For example, infants and children developing in different cultures show different patterns of face scanning (Geangu et al., 2016; Kelly et al., 2011; Senju et al., 2013), which are suggested to be adaptive to each of the cultural norms on the use of eye gaze (Argyle and Cook, 1976). Similarly, we recently demonstrated that sighted infants of blind parents (SIBPs), who experience qualitatively different eye gaze communication, show a distinct pattern of face scanning and gaze following, most notably from the second year of life (Senju et al., 2015). Adaptation to an individual’s particular social environment is fundamental for effective social learning and communication, as well as the formation of distinct cultural groups (Han et al., 2013). These findings are also consistent with the view that infants are born with initial predispositions to process their species-typical environment, which then also guide the later experience-dependent development of specialized cognition adaptive to the given individual environment (Johnson et al., 2015; Senju and Johnson, 2009). However, to date the evidence on this issue is limited to behavioural measures, and data is lacking on how and when processing in the infant brain is influenced by such variations in experience.

**Fig. 1:**
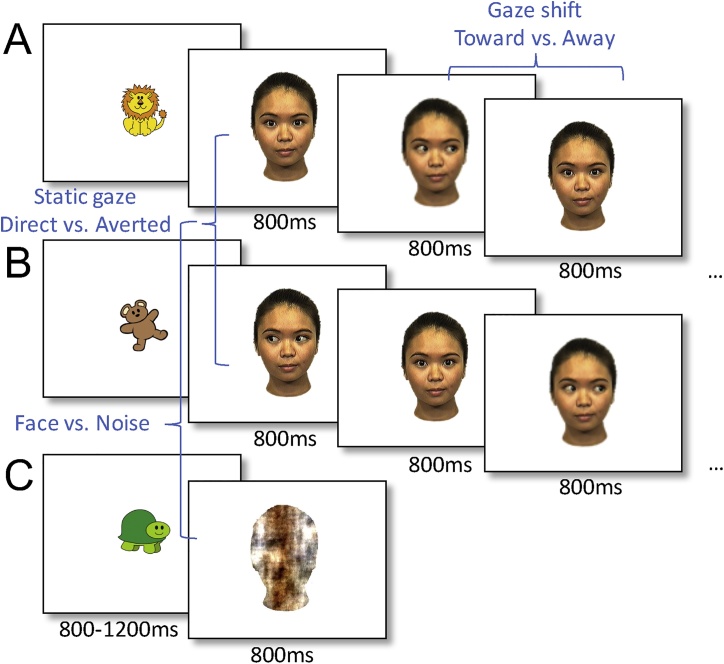
Schema of the ERP task consisting of three different types of trials (A. *Face* trials starting with direct gaze followed by gaze shifts, B. *Face* trials starting with *Averted* gaze followed by gaze shifts, C. *Noise* trials). The three different contrasts: static gaze (*Direct* vs. *Averted* gaze), gaze-shift (*Toward* vs. *Away* gaze) and *Face* vs. *Noise* are depicted in blue.

The current study is the first to investigate the role of eye gaze communication experience on the neural sensitivity for gaze processing. We tested 14 SIBPs at the age of 6–10 months of age, all of whose primary caregivers do not use typical forms of eye gaze communication because their visual impairment prevents them from seeing their babiesʼ eyes during face-to-face communication. Electroencephalography was used to record brain activity while SIBPs observed dynamic gaze shifts in a face image that moved either *Toward* or *Away* from the observer, presented on a video monitor (Fig. 1). From the recording, event-related potentials (ERPs) were analysed for posterior channels, which are known to show differences for the perception of different directions of gaze (Elsabbagh et al., 2009; Farroni et al., 2002) and gaze shift (Elsabbagh et al., 2012) in young infants. SIBP ERPs were then compared to the ERPs of 45 control infants of sighted parents (CTRLs), who participated in a separate study using the same paradigm, equipment and with experimenters similarly trained within the same research centre (Elsabbagh et al., 2012). The SIBP group also participated in a series of eye-tracking tests and the assessment of general social and cognitive skills at the time of testing (Senju et al., 2015), and was followed-up at 36 months of age to examine whether they show long-term typical development.

## 2 Methods

### 2.1 Participants

Fourteen sighted infants (6 males, mean age = 8.84 months; SD = 1.10) of blind parents (SIBP group) participated in the study. An additional SIBP child was excluded from the analyses due to not having a minimum of 10 valid trials in each contrast (see Supplementary information, Section 1 (SI-1), Table S1 for further details). All the blind parents were the primary caregivers of the infants, had visual impairment for at least 15 years prior to the testing, and could not see the infantsʼ eyes and gaze from the distance of 50 cm, based on self report (see SI-2, for more information on the level of visual impairment of the parents and the SIBP’s exposure to sighted adults). The ERP data were collected as part of a larger protocol, which also included a series of eye-tracking studies as well as standardised assessments of social and cognitive development (Senju et al., 2015). The data were then compared with the existing dataset of 45 infants with sighted parents (CTRL group, 15 males, mean age = 7.62 months; SD = 1.17), who originally participated in the British Autism Study of Infant Siblings (BASIS, a UK collaborative network examining infants at risk for autism (Elsabbagh et al., 2012)).

Eleven SIBP infants were also followed up at 36 months of age and were administered several behavioural assessments of social communicative and cognitive development: Mullen Scales of Early Learning (MSEL; Mullen, 1995), Vineland Adaptive Behaviour Scale (VABS; Sparrow et al., 2005), Autism Diagnostic Observation Scale-Generic (ADOS-G; Lord et al., 2000), Autism Diagnostic Interview-Revised (ADI-R; Lord et al., 1994) and Social and Communication Questionnaire (SCQ; Rutter et al., 2003) (see the participants characteristics in SI-3, Table S2). All SIBP infants but one obtained ADOS scores below the ADOS cut-off. One child did score above the cut-off for autism spectrum disorder (ASD), and subsequent to the research assessment, received a community clinical diagnosis of ASD.

### 2.2 Material and procedure

The task consisted in the presentation of four different female faces (face: 21.3° × 13.9°, eye: 1.6° × 2.7°) in the centre of a screen. A trial began with the presentation of a colourful picture of 1.6° × 1.6° for a variable duration of 800–1200 ms to attract infants’ attention. Then, a static face with *Direct* or *Averted* gaze was presented for 800 ms, followed by 3–6 gaze shifts from the same face (*Away* or *Toward* the viewer, Fig. 1) presented every 800 ms. As well as static faces and gaze shifts (*Face* trials), scrambled faces (*Noise* trials) were presented for 800 ms. Twelve scrambled faces were constructed from the same face stimuli (*Direct* gaze, left *Averted* gaze, right *Averted* gaze) for each female face, with randomization of the phase spectra while keeping constant the amplitude and colour spectra (Halit et al., 2004). The presentation of *Face* and *Noise* trials was pseudo-random such that 1) the same identity was used within the *Face* trials 2) which consisted in the intermittence of gaze shifts with opposite directions, and 3) the *Noise* trials were set to appear for one third of the total number of trials (Fig. 1). The faces were aligned with the centre of the screen so that the eyes appeared at a location where the fixation stimuli had been presented. All participants sat on their parents’ laps in front of a 40 × 29 cm screen at a distance of 60 cm. The infants’ gaze and movements were video-recorded.

### 2.3 EEG recording and ERPs extraction

A 128 electrodes Hydrocel Geodesic Sensor Net (Electrical Geodesics, Inc., USA) was used to record the EEG signal sampled at 500 Hz. Three infant event-related potentials (ERPs), P1, N290, and P400, known to be influenced in a number of face-perception tasks (de Haan et al., 2003; Halit et al., 2004), were extracted. The EEG signal was band-pass filtered (0.1–100 Hz), segmented 200 ms before and 800 ms after stimulus onset for each trial, and baseline corrected using a period of 200 ms before the stimulus onset. Automatic and manual (visual inspection) artefact rejection procedures were used to remove trials when the infants were not fixating the centre of the screen at stimulus onset, produced gaze shifts or head movements, and/or blinked, during the 800 ms period following onset of the face stimulus or gaze shift. The missing data from 12 or fewer channels were interpolated, otherwise the entire trial was rejected. The trials were then re-referenced to the average. Across all contrasts, the three ERPs were extracted following a previous study completed with the control data (Elsabbagh et al., 2012), over selected occipital channels and temporal windows where the task dependent characteristic waveform was observed (see SI-4, Fig. S1 and Table S3).

### 2.4 Analyses

The amplitude and latency of the three different event-related components of interest P1, N290 and P400 (de Haan et al., 2003), which have been identified in infants as precursors of the face-sensitive ERP component N170 observed in adults (Bentin et al., 1996), were analysed for each group to assess whether these components were differently modulated by the gaze shift direction (*Toward* vs. *Away* from the observer). A generalized linear model was conducted, with the Contrast gaze shift (*Toward* vs. *Away*) as a repeated-measures factor, Group (CTRL vs. SIBP) as between-subjects factor and Chronological age as a covariate. When the Contrast × Group interactions were significant, post hoc analyses were performed for each group of infants using *t*-tests. When the assumption of normal distribution was not met, follow-up analyses with non-parametric tests were conducted when necessary to corroborate the parametric analyses (see SI-8). Across all contrasts, infants who produced a minimum of 10 valid trials per condition were included in the analysis. The average number of trials recorded in each condition, the average number of valid trials after artefact rejection, and the number of infants included in the subsequent analyses are shown in SI-1, Table S1. ERPs for the contrast static faces with *Direct* versus *Averted* gaze direction (i.e. the first gaze direction that preceded the sequence of gaze shifts in a *Face* trial), as well as the *Face* versus *Noise* contrast were also analysed (see SI-5, Figs. S1 and S2). We also ran a follow-up ANCOVA including ADOS and Mullen ELC scores as additional covariates (see SI-6, Table S4). Finally, an additional bootstrap analysis of the distribution of the gaze shift effect in the CTRL group was conducted to assess whether the small sample size of the SIBP group and therefore the potential lack of power may have prevented the observation of a gaze effect in this group.

## 3 Results

**Fig. 2:**
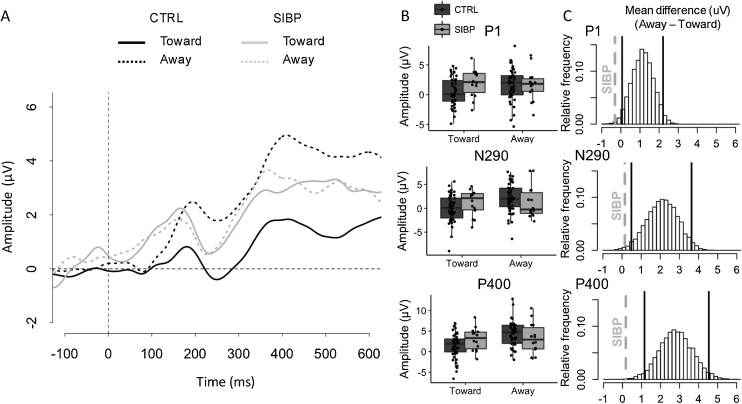
A) ERP waveforms for gaze shift *Toward* and *Away* for SIBP and CTRL groups over the occipito-temporal channels selected for this contrast (see SI-4, Fig. S1 for the precise location of the channels); B) Distributions of the amplitude of P1, N290 and P400 for both gaze shifts (*Toward* and *Away*) in each group (CTRL and SIBP). The boxplots depict the 25^th^, 50^th^ (median) and 75^th^ percentiles; C) Histograms depicting 10,000 bootstrap resamplings of the mean difference (*Away* – *Toward*) of the amplitude of P1, N290 and P400 between the conditions *Toward* and *Away* in the CTRL group (n = 14 resampled subjects). The mean differences of amplitude of P1, N290 and P400 in the SIBP group (grey dashed line) fall outside the 95% confidence intervals of the CTRL group (black lines).

The analyses revealed that the amplitudes of the components P1, N290, and P400 were differently modulated by the perceived direction of gaze shift between the SIBP and CTRL groups (significant interactions Group × Contrast Gaze shift for the amplitude of P1 (F(1,56) = 4.59, p = .036, η_p_^2^ = 0.08), N290 (F(1,56) = 5.13, p = .027, η_p_^2^ = 0.08) and P400, (F(1,56) = 8.40, p = .005, η_p_^2^ = 0.13). Post hoc tests revealed that the CTRL group showed smaller amplitudes of P1 (t(44) = 2.97, p = .005, d = 0.44), N290 (t(44) = 3.90, p < .001, d = 0.58) and P400 (t(44) = 4.89, p < .001, d = 0.75) for a gaze shift *Toward* than *Away* from the observer. By contrast, the amplitude of these components did not differentiate between the dynamic gaze directions in the SIBP group (all t (13) <0.38, all p > .712, all d < 0.10). A similar pattern was observed for P1 latency, which was shorter for gaze shift *Toward* than *Away* in CTRLs (t (44) = 3.67, p = .001, d = 0.55), but not in SIBPs (t (13) = 0.60, p = .561, d = 0.16) (Fig. 2A and B). However, the latter result should be treated with caution, as the Group × Contrast Gaze shift interaction was only marginal (F (1,56) = 3.92, p = .053, η_p_^2^ = 0.07; see SI-7, Fig. S4 for full results of latency analyses). Additionally, to examine whether the small sample size of the SIBP group may have prevented the observation of a gaze effect in this group due to lack of power, a bootstrap analysis (10,000 resamplings) of the mean difference of amplitude between the gaze shifts *Toward* and *Away* for P1, N290 and P400 in the CTRL group was performed. Fourteen subjects were randomly sampled (with replacement) from the CTRL group in each bootstrap to match the sample size of the SIBP group. The bootstrap analysis revealed that the mean difference (*Away*-*Toward*) of the SIBP group falls outside the distribution of the mean differences of amplitude of all three components in the resampled CTRL group. These results corroborate our previous analyses showing the absence of a gaze effect in the SIBP group (Fig. 2C).

Note that the group differences in the pattern of ERPs were restricted to the perception of dynamic gaze shifts, and were not identified when infants observed faces with static gaze, or when the ERPs for face perception were contrasted against those for non-facial *Noise* images (see additional ANOVAs in SI-5, Figs. S2 and S3 and bootstrapping analyses in SI-10, Figs. S5 and S6). However, the ANCOVA analyses with chronological age, Mullen ELC scores at 6–10 month and ADOS composite scores at 36 month as covariates revealed that in the Static and the Face vs. Noise contrasts, the latency for N290 was shorter for Direct vs. Averted gaze (Static: F(1,35) = 7.57, p = 0.009, η_p_^2^ = 0.18; Face vs. Noise: F(1,35) = 5.78, p = 0.021, η_p_^2^ = 0.13, see SI-6, Table S4). These results are in line with previous findings on face processing in infancy showing a greater N290 amplitude for faces than scrambled face in 4-month-old infants (Halit et al., 2004). Furthermore, we previously confirmed that at 6–10 months of age, the SIBP group shows similar patterns of face scanning compared to typically developing infants, do not differ in their social communication and show an overall high level of general development (Senju et al., 2015), see also SI-3, Table S2 and SI-6, Table S4). Thus, it is highly unlikely that the difference in ERP response to gaze shift can be explained by the different pattern of face scanning during the task, or more global impairment in social or cognitive development. We also analysed whether the individual differences in the amount of exposure to sighted adults, as well as the level of visual impairment of the parents, would affect the ERP response within the SIBP group, and did not find any significant association (see SI-2).

We followed up the SIBP group at 36 months of age and assessed if they manifested symptoms of ASD using the Autism Diagnostic Observation Scale Generic (ADOS-G; Lord et al., 2000) and the Autism Diagnostic Interview Revised (ADI-R; Lord et al., 1994), as the pattern of ERP response to dynamic gaze shift of the SIBP group resembled that of a group of infants who were later diagnosed with autism spectrum disorder (Elsabbagh et al., 2012). None of the SIBP infants who also participated in the follow-up assessment (n = 11) scored above the cut-off points for ASD on the ADOS or the ADI, except one child who scored above the cut-off points on the ADOS. After the research assessment, this child received a community diagnosis of ASD. The results did not significantly change when the data from this child was removed from the analysis (see SI-9).

## 4 Discussion

Functional neuroimaging of sighted infants with blind parents gave us the first opportunity to assess the impact of eye gaze communication experience on the development of neural specificity for gaze processing in young human infants. The results demonstrate that the differential ERP response to different direction of dynamic gaze shift, which has been observed in infants of sighted parents (Elsabbagh et al., 2012), requires typical experience of eye gaze communication with the primary caregiver. This experience is reduced in the SIBP group due to the visual impairment of their parents. Importantly, this effect was specific to eye gaze processing, and did not generalise to basic face processing or overall social and cognitive development (Senju et al., 2015; see SI-3, Table S2).

The current findings may be consistent with the notion of perceptual narrowing (Maurer and Werker, 2014) or with a degree of specialisation over time in eye gaze processing in which infantsʼ categorical perception becomes attuned to the category of social stimuli that they are most frequently exposed to. Perceptual narrowing has been shown with stimuli such as the faces of infants’ own species (Pascalis et al., 2002), own race (Liu et al., 2011; Wheeler et al., 2011), phonemes of their native language (Kuhl et al., 2003) and, as shown in the current study, the quantity of eye contact with their primary caregiver. Our findings, however, suggest that it is the communicative nature of interactive experience, not a mere exposure to the social stimuli that may contribute to the specialisation of functional brain development to eye gaze processing. The majority of SIBPs have had ample opportunity to observe the eyes of their primary caregiver, and the level of the parent's visual impairment did not affect the ERPs within the SIBP group. What was consistently different between groups was the interactive and contingent reciprocity of eye gaze communication with their parents, which seems to have contributed to the differential tuning to gaze processing of the SIBPs' brain. Our findings also resonate with a previous report that active experience of social interaction, rather than a mere passive exposure, contributes to the perceptual narrowing for native language (Kuhl et al., 2003), as well as another recent infant study demonstrating that infants' preference for native language speakers is based on the expectation of informative learning opportunities (Begus et al., 2016). However, it is unclear whether the SIBP group had the ability to differentiate gaze shifts (toward and away) at some point earlier in their development, or whether this ability had not developed as it does in typical development by 6–10months of age. Further studies will be essential to examine the earlier developmental trajectory of the SIBP to test if and when the perceptual narrowing takes place for eye gaze processing.

The pattern of neural responses to perceived dynamic gaze shifts in SIBP resembled that previously reported in a group of infants who were diagnosed with ASD later in their development (Elsabbagh et al., 2012). The current results might seem to be in conflict with the suggested link between the atypicality in this infant ERP response and later emergence of ASD, as only one infant in the SIBP group went on to develop ASD. However, we hypothesise that both sets of findings implicate a common neurodevelopmental process; that the cortical specialisation for gaze processing depends on adequate experience of typical gaze communication with adults. This factor can be compensated for by different sensory and communication channels in SIBP, or may be disturbed by an atypical neurodevelopmental trajectory due to genetic and/or epigenetic factors in infants who later develop ASD. Future studies will benefit from investigating whether early intervention for parenting behaviour targeting parent child social communication interaction (Green et al., 2017; Pickles et al., 2016), or a more targeted intervention for eye gaze processing (Murza et al., 2016) could rescue this neural marker for eye gaze processing of children with ASD.

An additional ANCOVA, which included ADOS and Mullen ELC as additional covariates, did not find significant group × gaze interaction in P1 and N290 amplitudes, while showing significant interaction on P400 (see SI). It could be claimed that this is consistent with Elsabbagh et al. (2012), who showed that the P400 is the most robust marker to differentiate those infants later diagnosed with ASD, and the impact of different early experience of eye gaze communication. It might also suggest that group differences in P1 and N290 could in part be attributed to the differences observed in ADOS and Mullen ELC scores. However, the direction of the group differences in these scores were actually *opposite* to those reported in Elsabbagh et al. (2012) for their ASD group: in our study, the SIBP group showed *lower* ADOS scores (i.e. fewer autistic traits) and *higher* Mullen ELC scores (i.e. more advanced overall development) than infants in the CTRL group. These scores seem to be linked to the reduction of ERP amplitude differences for gaze shift perception in the SIBP group, just as in the ASD group who showed higher ADOS scores and lower Mullen ELC scores. Further studies are needed to investigate the contribution of autistic traits and overall development on gaze processing, and how it interacts with diverse social and communicative experience.

It is also worth noting that the different ERP patterns for perceived gaze shift in SIBP, which we observed at 6–10 months of age, seem to precede in development the atypicalities in gaze processing behaviour, such as face scanning and gaze following, which was most prominent at the age of 12–15 months of age in SIBP (Senju et al., 2015). This finding mirrors those reported for infants at high risk for ASD, which also found a similar developmental sequence (Elsabbagh et al., 2012) with neural markers preceding overt behavioural indicators (Bedford et al., 2012). It is thus possible that the lack of ERP response we observe before the first birthday could be a developmental precursor of gaze processing behavioural differences between SIBPs and CTRLs emerging from the second year of life and later. Future studies will be needed to investigate whether this infant ERP response predicts the development of later social cognitive skills, beyond symptoms of ASD.

The study is not free from limitations, mainly due to the difficulty in recruiting this target population. Firstly, the small sample size of the SIBP population makes the study underpowered for investigating the impact of within-group variability on the ERP response, such as the amount of contact with sighted adults or level and nature of parents' visual impairment. Although the bootstrap analysis corroborates our findings of a lack of gaze effect in the SIBP population, we are cautious about the interpretation of the earlier components (P1 and N290), because of the small sample size, and relatively small effect sizes (η_p_^2^ = 0.08) compared to the medium to large effect size observed for P400 (η_p_^2^ = 0.13), and the possibility that they could be partly modulated by autistic traits or overall development. Again, this is consistent with Elsabbagh et al. (2012), who found that the P400 was the most robust marker to differentiate infants who were later diagnosed with ASD. Secondly, as we could only test a fairly wide age range (6–10 months), we were not able to assess the developmental trajectory of this ERP response in SIBP population in more detail. Although challenging, future studies with larger sample sizes and with a more refined longitudinal design will help us understand more precisely the developmental trajectory of eye gaze processing in this population.

To conclude, this study is the first to show that reduced early experience of non-verbal communication such as eye gaze affects the neural processing of eye gaze within the first year of life. It highlights the plasticity in human functional brain development, which adapts to the individual’s unique social experience and tunes it to the relevant signals for social communication and learning.

## Author contributions

AS, TC and MHJ designed research; AV, NG, LT performed research; AV analysed data; AV, AS wrote the paper and AV, AS, TC, MHJ, NG revised the paper.

## Conflict of Interest

None.
