# Supplementary material for: Infant neural sensitivity to eye gaze depends on early experience of gaze communication
Source: Dev Cogn Neurosci. 2018 May 26;34:1–6. doi: 10.1016/j.dcn.2018.05.007 (PMC6252267; doi:10.1016/j.dcn.2018.05.007)
Supplement: Supplementary file 1 [file mmc1.docx]

**Supplementary Information**

[1. Number of trials recorded and included in the analyses (Table S1). 2](#_Toc512826488)

[2. Level of visual impairment of the parents and amount of exposure to sighted adults. 2](#_Toc512826489)

[3. Participant characteristics (Table S2). 3](#_Toc512826490)

[4. Occipital channels and temporal windows selected for each contrast (Tables S3 and S4). 4](#_Toc512826491)

[5. Neural responses for static gaze and Face vs. Noise for SIBP and CTRL groups (Figures S2 and S3). 5](#_Toc512826492)

[6. ANCOVA of the neural responses for the three contrasts: static gaze, gaze shift and *Face* vs. *Noise* (Table S4). 7](#_Toc512826493)

[7. Analyses of the P1, N290 and P400 latencies for the gaze shift contrast (Figure S4). 8](#_Toc512826494)

[8. Non-parametric follow up analyses. 9](#_Toc512826495)

[9. Analyses of the neural responses for gaze shift for SIBP and CTRL groups without the SIBP participant with high ADOS score. 10](#_Toc512826496)

[10. Bootstrap analysis for the contrasts Static and Face vs. Noise (Figures S5 and S6). 11](#_Toc512826497)

# Number of trials recorded and included in the analyses (Table S1).

Table S1. Total number of trials produced in each contrast and condition, valid trials after artefact rejection, and number of infants included in the analyses. To be included in the analysis, infants had to have minimum 10 valid trials in each condition.

* Significant differences between the CTRL and SIBP groups for both the mean number of total trials (SIBP > CTRL, t (45) = 2.37, p = .022, d = .79) and the mean number of valid trials (SIBP > CTRL, t (45) = 2.27, p = .028, d = .76) for the stimuli *Noise*. All other pairwise comparisons between the CTRL and SIBP groups were not significant (all p > .081).

# Level of visual impairment of the parents and amount of exposure to sighted adults.

We examined whether the level of visual impairment of the parents would affect the ERP response to the gaze shift contrast (*Toward* vs. *Away*) within the SIBP group. The level of visual impairment of the SIBP parents was characterised by three different factors: the family type (only the mother / both parents are visually impaired), the mother’s ability to perceive the light (yes / no; here-after light perception), and the mother’s ability to perceive the orientation of someone’s head (yes / no; here-after orientation perception). Separate generalized linear models with level of impairment of the parents (family type, light perception or orientation perception) as between-subject factor, and gaze shift contrast as a repeated-measures factor were conducted on the amplitudes of the three ERPs components. The analyses revealed no effect of the level of visual impairment for the amplitudes of P1 and N290 (all F < 1.46, all p > .250) except for a non-significant trend for the amplitude of P400 (F (1,12) = 4.17, p = .064, η_p_^2^ = .26). This latter trend was characterised by higher P400 amplitude for both *Toward* and *Away* stimuli when both parents were visually impaired compared to when only the mother was blind. No main effect of gaze shift (all F < .16, all p > .700) or gaze shift x level of impairment interaction (all F < 2.18, all p > .166) were significant for the amplitudes of P1, N290 and P400 in the SIBP group. We also examined whether individual differences in the amount of exposure to sighted adults (characterised by the number of hours spent per week in the presence of sighted adults) would affect the ERP response to the gaze shift contrast within the SIBP group. Correlational analyses were conducted between the amount of exposure to sighted people and the amplitude difference between the gaze shifts *Toward* and *Away* for the three ERPs components. The analyses revealed a non-significant trend of negative correlation for N290 (r = -.52, p = .059, N = 14), and no significant correlation for either P1 (r = -.39, p = .172, N = 14), or P400 (r = -.43, p = .122, N = 14).

# Participant characteristics (Table S2).

Table S2. Participant’s characteristics at 6-10-month and 36-month visits.

*CA: Chronological age in months at the 6-10-month and 36-month visits. Mullen Scales of Early Learning Composite Standard Score; VABS: Vineland Adaptive Behaviour Scales (Communication, Daily Living Skills, Socialisation, Motor skills, Adaptive Behaviour Composite standard scores); AOSI: Autism Observation Scale for Infants; ADOS: Autism Diagnostic Observation Schedule (Total score and Comparison score); ADI: Autism Diagnostic Interview (Social Interaction, Communication Verbal and Stereotyped Behaviours and Restricted Interests algorithm scores); Social and Communication Questionnaire.*

* Significant differences were found between the CTRL and SIBP groups in chronological age (SIBP > CTRL, t (57) = 3.43, p = .001, d = 1.05, 6-10-month visit), Mullen ELC scores (SIBP > CTRL, t (57) = 4.16, p < .001, d = 1.27, 6-10-month visit), ADOS total scores (SIBP < CTRL, t (52) = 1.92, marginal p = .061, d = .65, 36-month visit) and ADOS composite scores (SIBP < CTRL, t (52) = 2.06, p = .044, d = .70, 36-month visit). All other pairwise comparisons between the CTRL and SIBP groups were not significant (all p > .125). Note that lower ADOS scores in SIBP group show fewer autistic traits in this group.

# Occipital channels and temporal windows selected for each contrast (Tables S3 and S4).

Figure S1. Occipital channels selected for each contrast. EEG was recorded using a 128-electrode Hydrocel Geodesic Sensor Net (Electrical Geodesic Inc., Eugene, OR, USA).

Table S3. Temporal windows for each contrast during which the peaks of the three ERP components P1, N290, and P400 were observed.

Across all three contrasts: Static gaze (*Direct* vs. *Averted*), Gaze shift (*Towards* vs. *Away*) and *Face* vs. *Noise*, the three ERP components analysed in the current study were observed over occipito-temporal electrodes. The electrodes were selected within this region of interest through visual inspection of the grand average for each component and each contrast globally (with no inspection of any possible contrast difference). Contrary to ERPs in the Static gaze and *Face* vs. *Noise* contrasts that are time-locked to the appearance of a stimulus, and thus result in larger amplitude waveforms, the response amplitude to gaze shifts is time-locked to the apparent motion of the eyes of the face and therefore temporal windows for this contrast differ from the temporal windows of the other two contrasts. Mean amplitudes for each subject were computed over the group of electrodes and time window selected for each contrast.

# Neural responses for Static gaze and Face vs. Noise for SIBP and CTRL groups (Figures S2 and S3).

For both the Static gaze (*Direct* vs. *Averted)* contrast and the *Face* vs. *Noise* contrast (Figure S2), the analyses revealed no significant main effect of contrast, no main effect of group, and no significant contrast x group interactions, for neither the amplitude, nor the latency of P1, N290 and P400 components [all F < 3.02, all p > .089] (Figure S2 and S3).

Figure S2. A) ERP waveforms for the Static gaze (*Direct* vs. *Averted*) contrast for SIBP and CTRL group over the occipito-temporal channels selected for this contrast (see SI-4, Figure S1 for the precise location of the channels); B) Distributions of the amplitude and latency of P1, N290 and P400 for both *Direct* vs. *Averted* in each participant group (CTRL and SIBP). The boxplots depict the 25th, 50th (median) and 75th percentiles.

Figure S3. A) ERP waveforms for the *Face* vs. *Noise* contrast for SIBP and CTRL group over the occipito-temporal channels selected for this contrast (see SI-4, Figure S1 for the precise location of the channels); B) Distributions of the amplitude and latency of P1, N290 and P400 for both *Face* vs. *Noise* in each participant group (CTRL and SIBP). The boxplots depict the 25th, 50th (median) and 75th percentiles.

# ANCOVAs of the neural responses for the three contrasts: Static gaze, Gaze shift and *Face* vs. *Noise* (Table S4).

Table S4. Analyses of the neural responses for the three contrasts (Static gaze, Gaze shift and *Face* vs. *Noise*) with chronological age, Mullen ELC scores at 6-10 month and ADOS composite scores at 36 month as covariates.

^a^ significant result compared to main analysis with only age as covariate, ^b^ non-significant result compared to main analysis with only age as covariate.

# Analyses of the P1, N290 and P400 latencies for the gaze shift contrast (Figure S4).

For the latency of P1, a marginal Contrast * Group interaction was observed [F (1,56) = 3.92, p = .053, η_p_^2^ = .07] and post hoc tests revealed that infants in the CTRL group showed a shorter latency of P1, for a gaze shift oriented *Toward* compare to a gaze shift oriented *Away* from the observer [t (44) = 3.67, p = .001, d = .77]. This effect was not seen in the SIBP group [t (13) = .60, p = .561, d = .21]. The analyses revealed no significant main effect of contrast, nor main effect of Group or Contrast x Group interaction for the latency of N290 [all F < .62, all p > .434] (Figure S4). For the latency of P400, a marginal effect of Contrast was observed [F (1,56) = 3.81, p = .056, η_p_^2^ = .06] as well as a significant Group x Contrast interaction [F (1,56) = 5.23, p = .026, η_p_^2^ = .09]. Despite the visual impression that the P400 latencies might differ between the gaze shifts *Toward* and *Away* in the SIBP group (Figure S1), the post hoc tests revealed no significant differences between *Toward* and *Away* gaze shifts in either the CTRL or the SIBP group [CTRL: t (44) = .53, p = .599, d = .11; SIBP: t (13) = 1.59, p = .137, d = .60] (Figure S4, top panel). However, the bootstrapping analysis for the gaze shift contrast shows that the mean differences of latency of P1 and P400 fall outside the resampled CTRL distribution with 14 subjects (Figure S4, bottom panel).


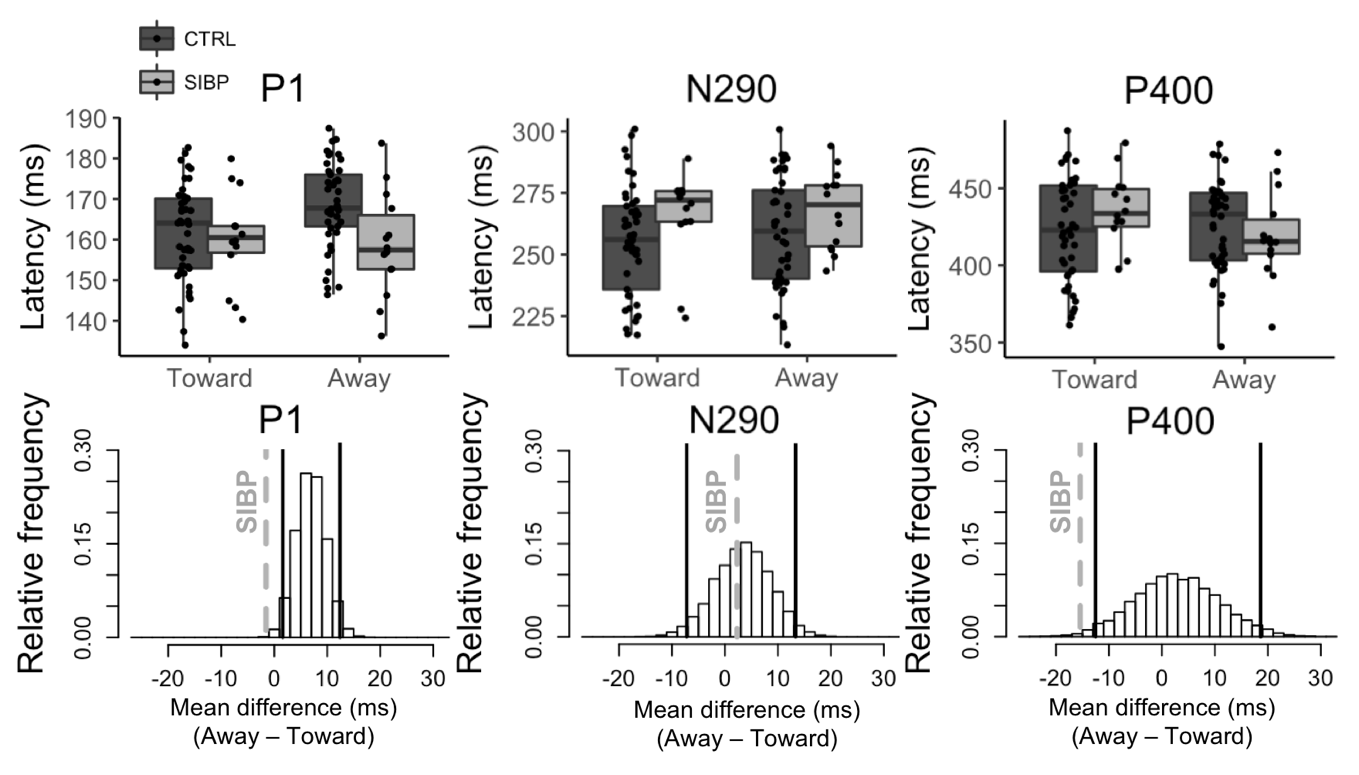


Figure S4. Top panel: Distribution of the latencies of P1, N290 and P400 for both gaze shifts (*Toward* and *Away*) in each participant group (CTRL and SIBP). The boxplots depict the 25th, 50th (median) and 75th percentiles; Bottom panel: Histograms depicting 10,000 bootstrap resamplings of the mean difference (*Away* - *Toward*) of amplitude of P1, N290 and P400 of 14 randomly selected subjects from the CTRL group. SIBP group in grey dashed lines. 95% confidence intervals of the CTRL group in black lines.

# Non-parametric follow up analyses.

The normality of the distribution of the amplitude and latencies of all ERPs (P1, N290 and P400) for all contrasts (Static gaze, Gaze shift and *Face* vs. *Noise*) was assessed with the Shapiro-Wilk test prior to analyses. The distribution of four subsets (listed below) did not meet the assumption of normality. Therefore, non-parametric tests were conducted when necessary to corroborate the parametric analyses.

- The distribution of the amplitude of the N290 to *Direct* gaze (in the contrast Static gaze) in the SIBP group did not meet normality (W (12) = .78, p = .006). Therefore, a Wilcoxon Signed Ranks test was conducted to analyse the contrast *Direct* vs. *Averted* gaze in this group. The Wilcoxon Signed Ranks test revealed no significant difference between the amplitude of the N290 to *Direct* vs. *Averted* gaze (Z = .24, p = 0.814). This result was also confirmed when excluding the SIBP participant who showed a high ADOS score (Z = .09, p = 0.929).
- The distribution of the latency of the P400 to *Averted* gaze (in the contrast Static gaze) in the CTRL group did not meet normality (W (32) = 0.92, p = .026). Therefore, a Wilcoxon Signed Ranks test was conducted to analyse the contrast *Direct* vs. *Averted* gaze in this group. The Wilcoxon Signed Ranks test revealed no significant differences between the latency of the P400 to *Direct* vs. *Averted* gaze (Z = .54, p = 0.588).
- The distribution of the amplitude of the N290 to gaze shift *Away* (in the contrast Gaze shift) in the SIBP group did not meet normality (W (14) = .87, p = .036). Therefore, a Wilcoxon Signed Ranks test was conducted to analyse the contrast gaze shift *Toward* vs. *Away* in this group. The Wilcoxon Signed Ranks test revealed no significant difference between the amplitude of the N290 of gaze shift *Toward* vs. *Away* (Z = .35, p = 0.730). This result was also confirmed when excluding the SIBP participant who showed a high ADOS score (Z = .38, p = 0.701).
- The distribution of the latency of the N290 to gaze shift *Toward* (in the contrast Gaze shift) in the SIBP group did not meet normality (W (14) = .77, p = .002). Therefore, a Wilcoxon Signed Ranks test was conducted to analyse the contrast gaze shift *Toward* vs. *Away* in this group. The Wilcoxon Signed Ranks test revealed no significant difference between the latency of the N290 of gaze shift *Toward* vs. *Away* (Z = .47, p = 0.638). This result was also confirmed when excluding the SIBP participant who showed a high ADOS score (Z = .25, p = 0.807).

# Analyses of the neural responses for Gaze shift for SIBP and CTRL groups without the SIBP participant with high ADOS score.

A secondary analysis of the SIBP and CTRL groups’ neural responses to gaze shift (*Toward* vs. *Away*) was conducted but without the SIBP participant who showed a high ADOS score. A generalized linear model, with the contrast Gaze shift as a repeated-measures factor, Group (CTRL vs. SIBP) as between-subjects factor and Chronological age as a covariate, replicated the main findings reported in the main article. The analyses replicated the main results, demonstrating that the amplitudes of P1, N290 and P400 were differently modulated by the perceived direction of gaze shift between SIBP and CTRL groups. Significant Group x Contrast Gaze shift interactions were found for the amplitude of P1 (F (1,55) = 4.00, p = .050, η_p_^2^ = .07), N290 (F (1,55) = 4.83, p = .032, η_p_^2^ = .08) and P400 (F (1,55) = 7.14, p = .010, η_p_^2^ = .12). As reported in the main text, the post hoc tests revealed that the CTRL group showed smaller amplitudes of P1, N290 and P400 for gaze shifts *Toward* than *Away* from the observer (all t (44) > 2.97, all p < .005). By contrast, in the SIBP group, the amplitude of these components did not differentiate between the two gaze shift directions (all t (12) < .32, all p > .757). Finally, the Group x Contrast Gaze shift interaction for the P1 latency reported in the main article did not reach significance when the SIBP participant with a high ADOS score was excluded (F (1,55) = 3.39, p = .071, η_p_^2^ = .06), again replicating the main results. However, explorative post hoc tests showed that the P1 latency was, as reported in the main article, shorter for gaze shifts *Toward* than *Away* in CTRLs (t (44) = 3.67, p = .001, d = .77), but not in SIBPs (t (12) = .50, p = .628, d = .20).

# Bootstrap analysis for the contrasts Static and Face vs. Noise (Figures S5 and S6).

Figure S5. Histograms depicting 10,000 bootstrap resamplings of the mean difference of amplitude (left column) and latency (right column) between the static faces *Direct* vs. *Averted* for P1, N290 and P400 in the CTRL group. Fourteen subjects of the CTRL group were randomly selected for each resampling. The mean differences *Averted*-*Direct* of the SIBP group are depicted in grey dashed lines. The 95% confidence intervals of the CTRL group are depicted in black lines.

Figure S6. Histograms depicting 10,000 bootstrap resamplings of the mean difference of amplitude (left column) and latency (right column) between the conditions *Face* and *Noise* for P1, N290 and P400 in the CTRL group. Fourteen subjects of the CTRL group were randomly selected for each resampling. The mean differences *Noise*-*Face* of the SIBP group are depicted in grey dashed lines. The 95% confidence intervals of the CTRL group are depicted in black lines.
